# Acute effects of empagliflozin on open-loop baroreflex function and urine output in streptozotocin-induced type 1 diabetic rats

**DOI:** 10.1186/s12576-024-00938-z

**Published:** 2024-09-28

**Authors:** Toru Kawada, Hiromi Yamamoto, Masafumi Fukumitsu, Takuya Nishikawa, Hiroki Matsushita, Yuki Yoshida, Kei Sato, Hidetaka Morita, Joe Alexander, Keita Saku

**Affiliations:** 1 Department of Cardiovascular Dynamics, National Cerebral and Cardiovascular Center, Osaka, 564-8565 Japan; 2 Department of Cardiovascular Medicine, Kurashiki Central Hospital, Ohara HealthCare Foundation, Okayama, 710-8602 Japan; 3 Department of Research Promotion and Management, National Cerebral and Cardiovascular Center, Osaka, 564-8565 Japan; 4 Medical and Health Informatics Laboratories, NTT Research, Inc, Sunnyvale, CA 94085 USA; 5 Bio Digital Twin Center, National Cerebral and Cardiovascular Center, Osaka, 564-8565 Japan

**Keywords:** Sodium–glucose cotransporter 2, Sympathetic nerve activity, Arterial pressure, Urine flow, Equilibrium diagram

## Abstract

Although sympathetic suppression is considered one of the mechanisms for cardioprotection afforded by sodium–glucose cotransporter 2 (SGLT2) inhibitors, whether SGLT2 inhibition acutely modifies sympathetic arterial pressure (AP) regulation remains unclear. We examined the acute effect of an SGLT2 inhibitor, empagliflozin (10 mg/kg), on open-loop baroreflex static characteristics in streptozotocin (STZ)-induced type 1 diabetic and control (CNT) rats (*n* = 9 each). Empagliflozin significantly increased urine flow [CNT: 25.5 (21.7–31.2) vs. 55.9 (51.0–64.5), STZ: 83.4 (53.7–91.7) vs. 121.2 (57.0–136.0) μL·min^−1^·kg^−1^, median (1st–3rd quartiles), *P* < 0.001 for empagliflozin and STZ]. Empagliflozin decreased the minimum sympathetic nerve activity (SNA) [CNT: 15.7 (6.8–18.4) vs. 10.5 (2.9–19.0), STZ: 36.9 (25.7–54.9) vs. 32.8 (15.1–37.5) %, *P* = 0.021 for empagliflozin and *P* = 0.003 for STZ], but did not significantly affect the peripheral arc characteristics assessed by the SNA–AP relationship. Despite the significant increase in urine flow and changes in several baroreflex parameters, empagliflozin preserved the overall sympathetic AP regulation in STZ-induced diabetic rats. The lack of a significant change in the peripheral arc may minimize reflex sympathetic activation, thereby enhancing a cardioprotective benefit of empagliflozin.

## Introduction

Sodium–glucose cotransporter 2 (SGLT2) inhibitors have been developed to treat diabetes mellitus (DM). After empagliflozin, an SGLT2 inhibitor, showed reduced cardiac events in patients with DM [1], the use of SGLT2 inhibitors has been expanded to treat heart failure and chronic kidney disease [2]. However, mechanisms involved in the cardiorenal protection by SGLT2 inhibitors are not fully understood, and sympathetic suppression may be one of the potential mechanisms [3, 4]. Sympathetic activation may be associated with renal stress derived from the burden of reabsorbing large amounts of glucose in DM [5]. SGLT2 inhibitors may relieve renal stress and reduce sympathetic nerve activity (SNA). In addition, SGLT2 inhibitors may directly suppress neurons in the rostral ventrolateral medulla [6], which is the center of sympathetic outflow.

In a previous study, we performed an open-loop baroreflex experiment on Goto–Kakizaki (GK) diabetic rats and tested whether empagliflozin acutely suppresses SNA [7]. Empagliflozin did not significantly modify SNA or sympathetic arterial pressure (AP) regulation despite a significant increase in urine flow (UF) and urine glucose excretion in GK rats. However, DM was moderate in GK rats, with non-fasted blood sugar (BS) levels < 300 mg/dL [208 (194–231) mg/dL, median (1st–3rd quartiles), *n* = 7]. We hypothesized that empagliflozin shows a significant hemodynamic effect in a DM model with higher BS. To test the hypothesis, we examined the acute effect of empagliflozin in streptozotocin (STZ)-induced type 1 diabetic rats, defining DM as non-fasted BS > 300 mg/dL and compared the results between STZ and control (CNT) rats.

## Methods

### Ethical approval

Male Wistar–Kyoto rats were purchased from Japan SLC. The rats were cared for in strict accordance with the Guiding Principles for the Care and Use of Animals in the Field of Physiological Sciences, which has been approved by the Physiological Society of Japan. The Animal Subjects Committee at the National Cerebral and Cardiovascular Center reviewed and approved the experimental protocols (17029, 21009).

### Induction of DM

Streptozotocin (FUJIFILM Wako Pure Chemical Corporation, Japan) was dissolved in physiological saline to a concentration of 20 mg/mL and injected intraperitoneally (70 mg/kg) to male Wistar–Kyoto rats under isoflurane inhalation anesthesia at 12–13 weeks of age. After 5–8 weeks, the rats underwent an acute experiment to assess the open-loop baroreflex static characteristics. At the beginning of the acute experiment, a blood droplet was obtained by puncturing the tail artery with a 27G needle under anesthesia, and a commercial glucose meter (FreeStyle, Freedom Lite, Nipro, Japan) was used to measure the BS level. The rats were considered to develop DM when non-fasted BS > 300 mg/dL.

### Acute baroreflex experiment

An acute baroreflex experiment was performed in CNT (*n* = 9) and STZ-induced DM rats (n = 10). The rats were anesthetized with an intraperitoneal injection (2 mL/kg) of a mixture of urethane (250 mg/mL) and α-chloralose (40 mg/mL). The anesthetic mixture was diluted 18-fold with physiological saline and infused continuously via the right femoral vein (2 mL·kg^−1^·h^−1^). Ringer’s lactate solution was constantly infused (4 mL·kg^−1^·h^−1^) for fluid maintenance. The rats were ventilated mechanically with oxygen-enriched air. An arterial line was inserted into the right femoral artery to measure AP. Heart rate (HR) was derived from an electrocardiogram using a cardiotachometer. A heating pad and a lamp maintained the rat’s body temperature between 37 °C and 38 °C.

For SNA recording, a postganglionic branch of the splanchnic sympathetic nerve was exposed retroperitoneally. A pair of stainless steel wire electrodes (AS633, Cooner Wire, CA, USA) was attached to the nerve and fixed with silicone glue (Kwik-Sil, World Precision Instruments, FL, USA). The electrical signal was band-pass filtered between 150 and 1000 Hz, full-wave rectified, and low-pass filtered at a cut-off frequency of 30 Hz. The noise level of SNA was determined after intravenously injecting a ganglionic blocker, hexamethonium bromide (60 mg/kg), at the end of the experiment.

The bilateral carotid sinus baroreceptor regions were isolated from the systemic circulation [8, 9], and a shaker (ET-126, Labworks, CA, USA) was used to externally regulate the carotid sinus pressure (CSP). Sectioning the aortic depressor and vagal nerves in the neck region minimized reflexes other than the carotid sinus baroreflex.

For urine sampling, the ureters were inserted with a polyethylene tube (KN-392-SP 8, Natsume, Japan) through a transverse laparotomy. Urine was collected in a vertically placed 1-mL syringe on the surgical table’s lateral side. Subsequently, urine volume (UV) was calculated from the hydrostatic pressure relative to that of 1 mL of physiological saline [10].

### Protocol

CSP was decreased to 60 mmHg for 5 min and then increased stepwise up to 180 mmHg in 20-mmHg increments per minute. The 11-min stepwise CSP input sequence was repeated and denoted as S1–S8. Empagliflozin (MedChemExpress, NJ, USA) was dissolved in dimethyl sulfoxide (DMSO) to a 10 mg/100 μL concentration and diluted with polyethylene glycol and physiological saline to a final concentration of 10 mg/mL (10% v/v DMSO, 45% v/v polyethylene glycol 200, and 45% v/v physiological saline). One minute after S2 was completed, the empagliflozin solution was administered intravenously at 10 mg/kg (1 mL/kg, bolus).

### Data analysis

Among ten STZ rats, SNA recording was unsuccessful in one STZ rat; hence, the open-loop baroreflex static characteristics were analyzed in nine CNT rats and nine STZ rats. The CSP, SNA, AP, HR, and UV data were stored on a laboratory computer system at 1000 Hz via a 16-bit analog-to-digital converter. In each of the S1–S8 sequences, the mean of SNA, AP, and HR values was obtained during the last 10 s of each step. Furthermore, the data were averaged for two consecutive sequences and referred to as periods of baseline (BL), T1, T2, and T3, respectively.

The SNA was normalized because the absolute SNA amplitude varied significantly across rats depending on recording conditions. The value after the ganglionic blockade was defined as 0%, and that corresponding to the CSP of 60 mmHg during BL was 100%.

A four-parameter logistic function was used to quantify the static characteristics of the baroreflex total arc (AP vs. CSP), HR control (HR vs. CSP), and neural arc (SNA vs. CSP) (Eq. 1) [11, 12]:

1 $$y=\frac{{P}_{1}}{1+exp\left[{P}_{2}\left(CSP-{P}_{3}\right)\right]}+{P}_{4}$$

where *y* is the output (AP, HR, or SNA); *P*_1_ is the response range (i.e., the difference between the minimum and maximum output values); *P*_2_ is the slope coefficient; *P*_3_ is the midpoint pressure on the CSP axis; and *P*_4_ is the minimum value of the sigmoid curve. The maximum gain (*G*_max_) was calculated as *P*_1_ × *P*_2_/4, which corresponds to the absolute value of the steepest slope on the sigmoid curve at CSP = *P*_3_.

The static characteristics of the baroreflex peripheral arc (AP vs. SNA) approximated a straight line, and linear regression was used to estimate its slope and intercept [12]. The baroreflex equilibrium diagram was used to identify the operating point of the carotid sinus baroreflex [13, 14]. Subsequently, the operating-point AP and SNA were determined from the intersection of fitted neural and peripheral arcs on the equilibrium diagram.

The UF (μL/min) was calculated from an increment of UV summed from both ureters. The normalized UF (nUF, μL·min^−1^·kg^−1^) was defined as the UF divided by the rat’s body weight. The AP–nUF relationship was examined using linear regression based on previous studies [7, 10, 15] and reported as the relationship for a single kidney by halving the measured nUF.

### Blood and urine samples

Glucose and creatinine concentrations in blood and urine were measured in six out of nine CNT rats and five out of ten STZ rats to gain insights into renal function. To prevent blood withdrawal from interfering with the assessment of open-loop baroreflex function, the blood sample was obtained from the arterial line after completing the step input protocol and before administering hexamethonium bromide. After centrifugation, the plasma sample was frozen at − 80 °C. Urine samples were obtained during BL, T1, T2, and T3 periods and frozen at − 80 °C. Subsequently, glucose and creatinine concentrations in the plasma and urine samples were measured by outsourcing (SRL Inc., Japan). Creatinine clearance (C_cr_) was calculated based on the mean nUF, assuming that the plasma creatinine concentration had not changed significantly during the experiment. The noradrenaline level in the plasma sample was measured using high-performance liquid chromatography system (Eicom, Japan) following an alumina adsorption procedure.

### Statistical analysis

Data were expressed as median and 1st–3rd quartiles (in parentheses) or as mean ± standard deviation (SD). The effects of empagliflozin and STZ were examined using a two-way analysis of variance (ANOVA) repeated on one factor (empagliflozin) modified for nonparametric comparisons using aligned rank transform [16, 17]. When the main effect of empagliflozin or the interaction effect was significant, the post-hoc analysis was conducted between the BL and T3 periods within the CNT or STZ group using the Wilcoxon signed-rank test. When the main effect of STZ or the interaction effect was significant, the post-hoc analysis was conducted between the CNT and STZ groups for the BL or T3 period using the Mann–Whitney test. The number of post-hoc comparisons was either 2 or 4, and the significance level was adjusted using Holm’s method [18].

Plasma glucose, creatinine, and adrenaline concentrations were compared between six CNT and five STZ rats using the Mann–Whitney test. Urine-related parameters (urine glucose concentration, urine creatinine concentration, average nUF, glucose excretion, and C_cr_ per kidney) during BL were also compared between the two groups using the Mann–Whitney test. Time-dependent changes in urine-related parameters within each group were analyzed using repeated-measures one-way ANOVA with the Greenhouse–Geisser correction, followed by Dunnett’s test (Prism 8, GraphPad Software, CA, USA). In all the statistical analyses, differences were considered significant at *P* < 0.05.

## Results

The weeks of age on the day of the acute experiment were not significantly different between both groups [CNT: 17.1 (16.8–21.7) vs. STZ: 19.0 (18.9–20.1) weeks, median (1st–3rd quartiles), *P* = 0.408 by the Mann–Whitney test]. The body weight was significantly lower in the STZ than in the CNT group [CNT: 400 (383–420) vs. STZ: 333 (320–347) g, *P* < 0.001 by the Mann–Whitney test].

**Fig. 1:**
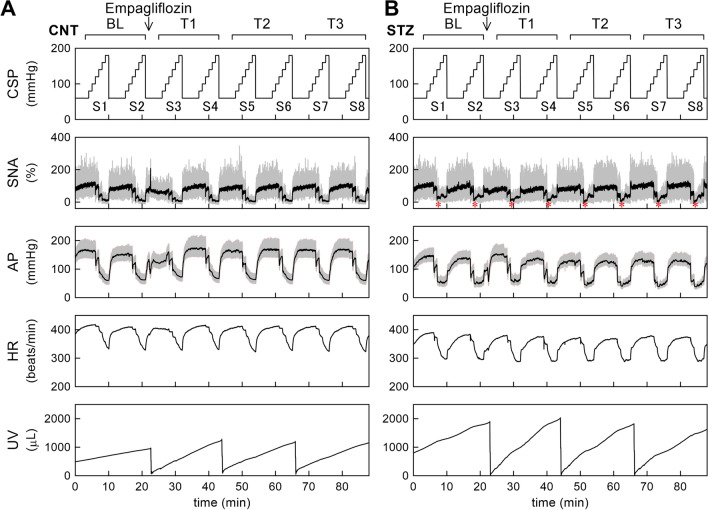
Time series obtained from a control (CNT) rat (**A**) and a streptozotocin (STZ)-induced diabetic rat (**B**). Carotid sinus pressure (CSP), sympathetic nerve activity (SNA), arterial pressure (AP), heart rate (HR), and urine volume (UV) during stepwise input sequences (S1–S8) are shown. In the CSP plot, the signal was displayed at 10 Hz. In the SNA plot, 10-Hz resampled (gray) and 2-s moving averaged (black) signals are shown. In the STZ rat, SNA showed nadirs in the CSP midrange (asterisks). In the AP plot, 100-Hz resampled (gray) and 2-s moving averaged (black) signals are shown. In the HR and UV plots, the data show 2-s moving averaged signals. The sudden drops in UV indicate manual urine removals. Empagliflozin (10 mg/kg) was administered 1 min after S2 completion. The data were analyzed for baseline (BL) and 1st (T1), 2nd (T2), and 3rd (T3) periods after empagliflozin administration

Figure 1 shows the time series of the acute experiment. In a CNT rat (Fig. 1A), a stepwise increase in CSP suppressed SNA and decreased AP and HR. Empagliflozin administration transiently decreased the maximum SNA and AP during S3, but these effects disappeared during S4 and onward. HR response did not show a significant change after empagliflozin administration. The bottom panel shows UV summed from both ureters. UV increased with time, and urine removals caused the abrupt drops. Empagliflozin steepened the increasing slope of UV, and the slope remained increased during T3 compared with BL.

In an STZ rat (Fig. 1B), a stepwise increase in CSP suppressed SNA and decreased AP and HR. A close look at the SNA plot indicated that SNA showed nadirs during the CSP midrange (asterisks). Empagliflozin administration slightly decreased the maximum SNA and increased the maximum AP during S3, but these effects subsided with time. The maximum HR slightly decreased after empagliflozin administration in this rat. The increasing slope of UV during BL was steeper in the STZ rat than in the CNT rat. Empagliflozin further steepened the increasing slope of UV, which continued until T3.

**Fig. 2:**
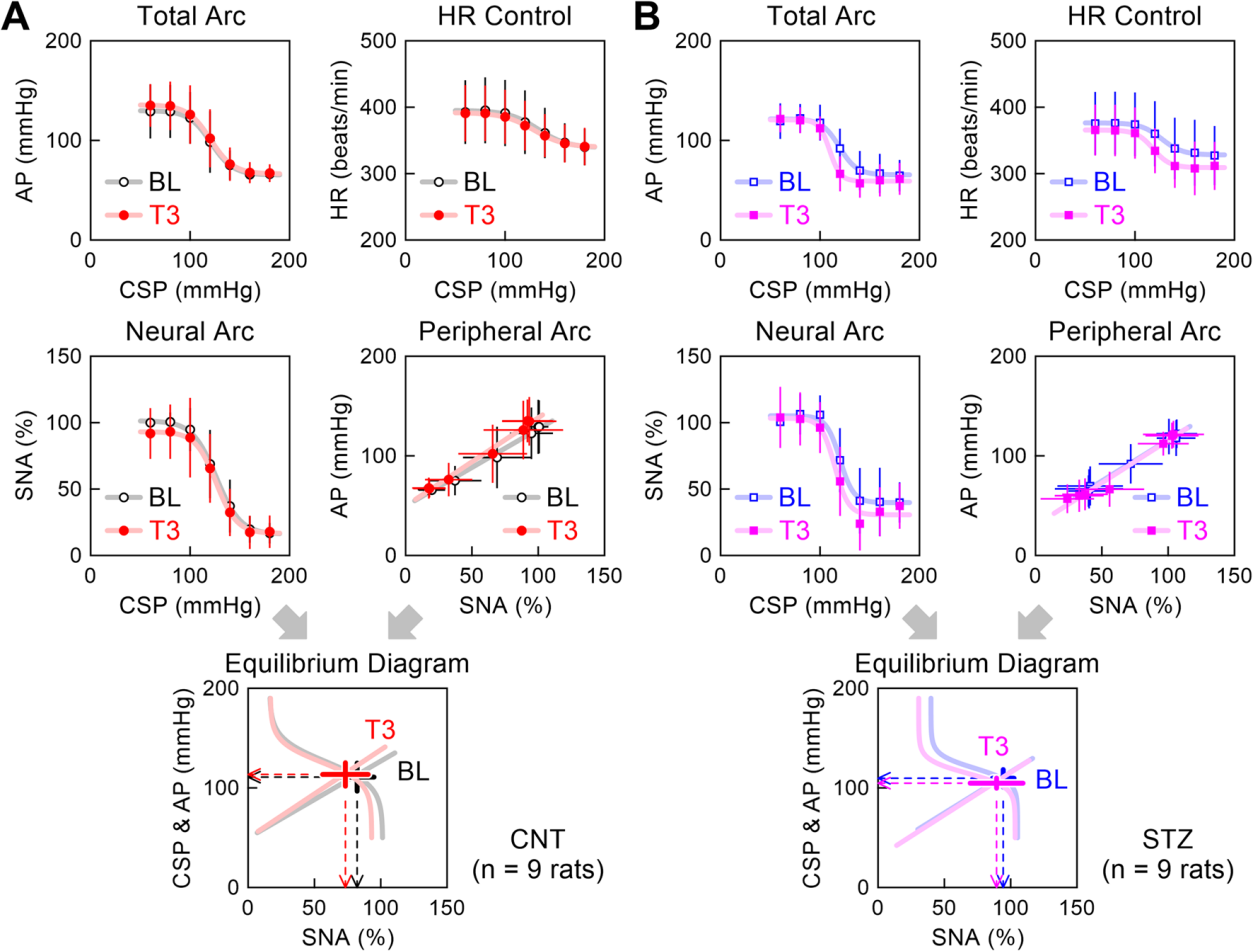
Pooled data showing the total arc, heart rate (HR) control, neural arc, and peripheral arc obtained from the control (CNT) group (**A**) and streptozotocin (STZ) group (**B**). The open and closed symbols indicate data during the baseline (BL) and third period (T3) after empagliflozin administration, respectively. Each data point shows the mean ± SD values. In the baroreflex equilibrium diagrams, the thick horizontal and vertical bars show the mean ± SD of sympathetic nerve activity (SNA) and arterial pressure (AP) of the operating point, respectively. CSP, carotid sinus pressure

Figure 2 shows pooled data of the open-loop baroreflex static characteristics. In the CNT group (Fig. 2A), the mean sigmoid curves of the total arc and HR control did not change between BL and T3, indicating no significant effect of empagliflozin. The neural and peripheral arcs were slightly displaced between BL and T3. In the baroreflex equilibrium diagram, the leftward and downward arrowheads indicate the means of the operating-point AP and SNA, respectively.

In the STZ group (Fig. 2B), empagliflozin slightly reduced AP during the CSP midrange without significantly affecting the minimum and maximum AP values in the total arc. The mean sigmoid curve of the HR control slightly shifted downward after empagliflozin administration. The neural arc showed a change similar to that in the total arc. The peripheral arc did not differ significantly between BL and T3.

**Fig. 3:**
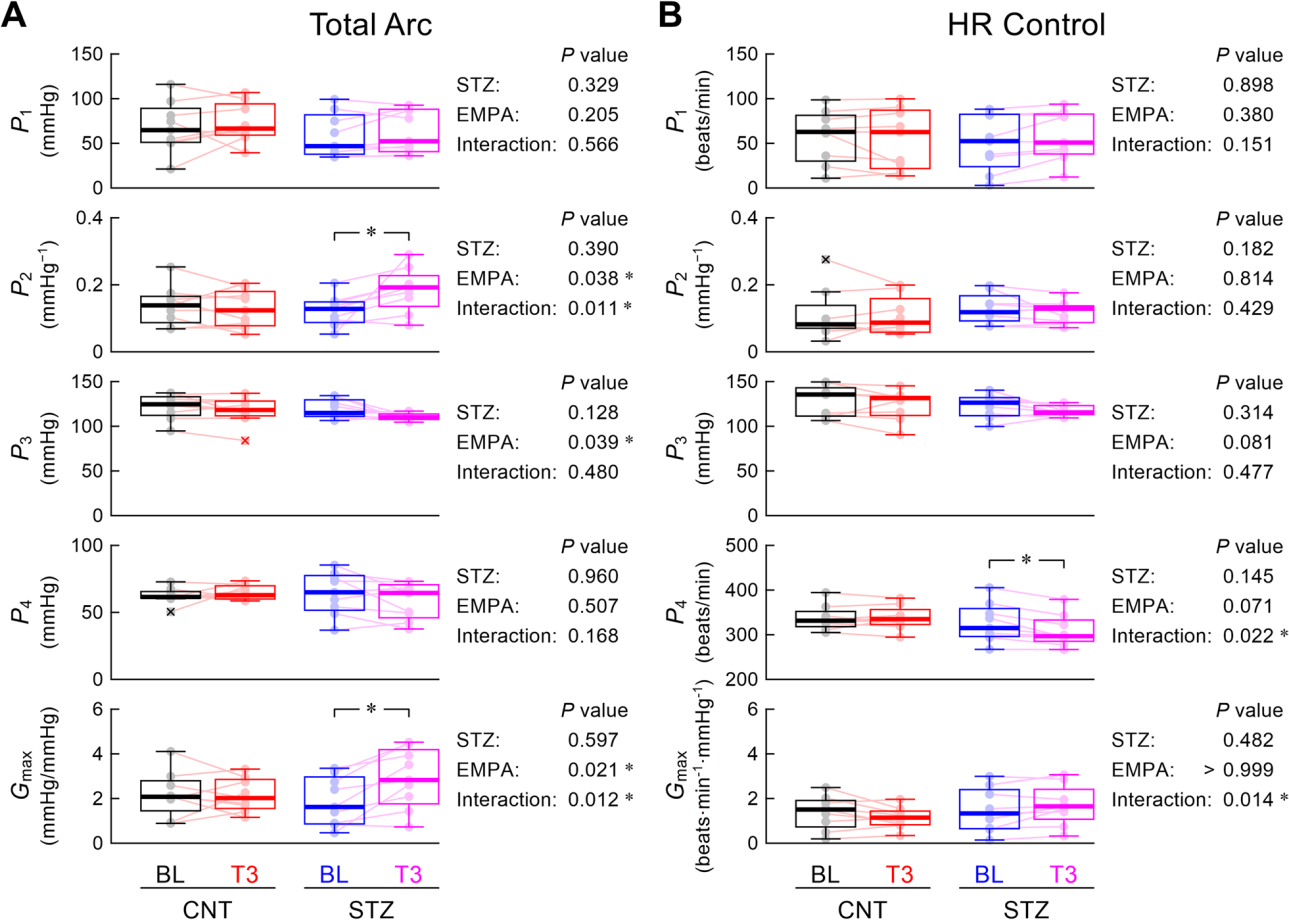
Box-and-whisker plots showing the parameters of the total arc (**A**) and heart rate (HR) control (**B**). The thick horizontal bar represents the median. The box indicates the 1st–3rd quartiles, whereas the whisker indicates the minimum and maximum values, excluding outliers. Outliers are depicted by a cross ( ×). The data points of respective rats are shown as pale small circles. In the right, *P*-values are shown for the effect of streptozotocin (STZ), the effect of empagliflozin (EMPA), and the interaction effect derived from a two-way analysis of variance with aligned rank transform. *P*_1_, response range; *P*_2_, slope coefficient; *P*_3_, midpoint pressure; *P*_4_, minimum value; *G*_max_, maximum gain; BL, baseline period; T3, third period after empagliflozin administration; CNT, control. The post-hoc analysis was performed using the Wilcoxon signed-rank test (BL vs. T3) or Mann–Whitney test (CNT vs. STZ), with an appropriate correction of the significance level based on Holm's method. * indicates *P* < 0.05

Figure 3 summarizes the statistical analyses of hemodynamic parameters. In the total arc (Fig. 3A), empagliflozin showed significant main effects for the slope coefficient (*P*_2_), midpoint pressure (*P*_3_), and maximum gain (*G*_max_). The significant interaction effect and post-hoc analysis suggest that empagliflozin increased *P*_2_ and *G*_max_ mainly in the STZ group. In the HR control (Fig. 3B), the significant interaction effect on the minimum value (*P*_4_) and post-hoc analysis indicate that empagliflozin decreased *P*_4_ mainly in the STZ group.

**Fig. 4:**
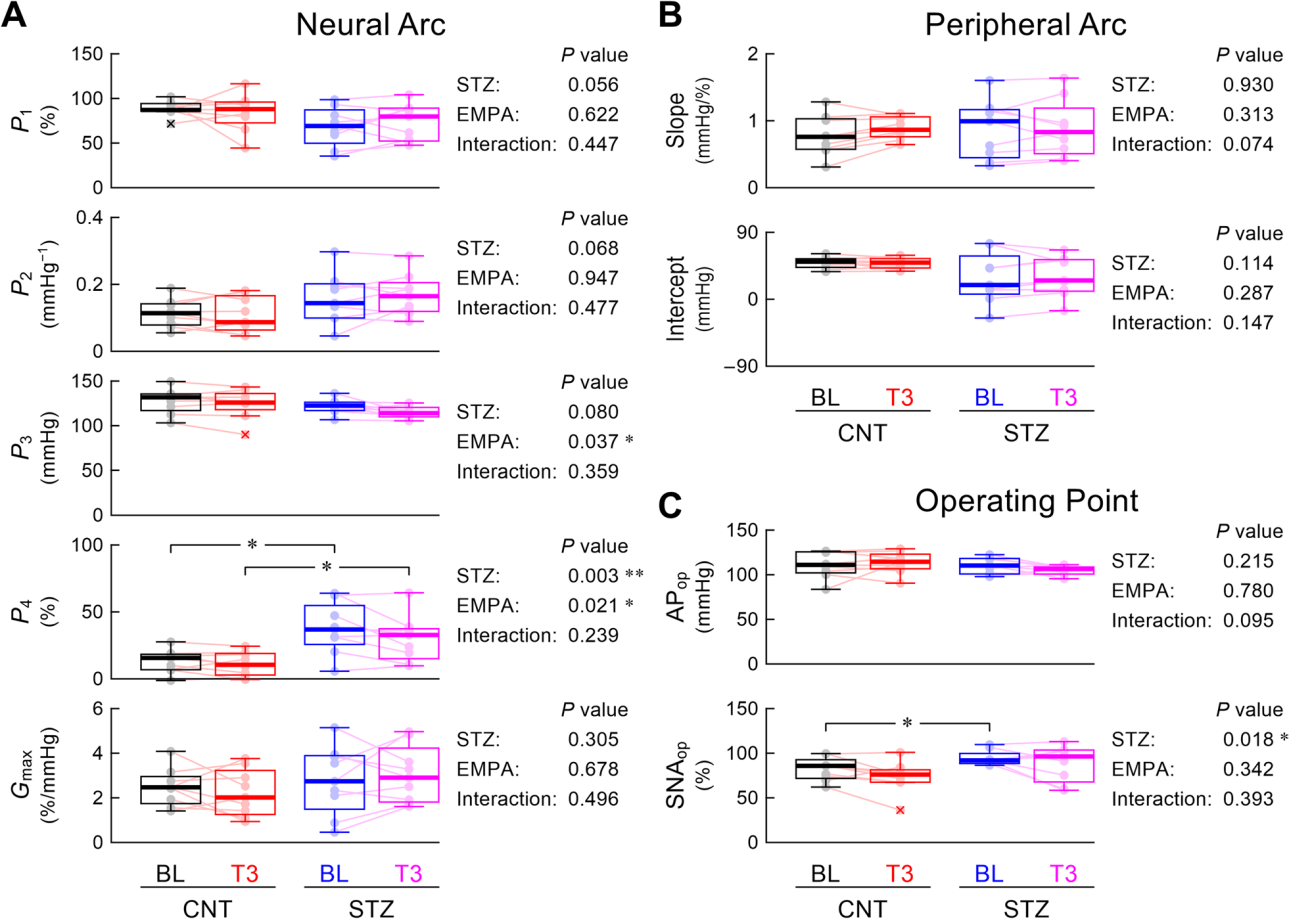
Box-and-whisker plots showing the parameters of the neural arc (**A**), peripheral arc (**B**), and operating point (**C**). In the right, *P*-values are shown for the effect of streptozotocin (STZ), the effect of empagliflozin (EMPA), and the interaction effect derived from a two-way analysis of variance with aligned rank transform. *P*_1_, response range; *P*_2_, slope coefficient; *P*_3_, midpoint pressure; *P*_4_, minimum value; *G*_max_, maximum gain; AP_op_, operating-point arterial pressure; SNA_op_, operating-point sympathetic nerve activity; BL, baseline period; T3, third period after empagliflozin administration; CNT, control. The post-hoc analysis was performed using the Wilcoxon signed-rank test (BL vs. T3) or Mann–Whitney test (CNT vs. STZ), with an appropriate correction of the significance level based on Holm’s method. * and ** indicate *P* < 0.05 and *P* < 0.01, respectively

Figure 4 summarizes the statistical analyses of parameters of the neural arc, peripheral arc, and operating point. In the neural arc (Fig. 4A), the response range (*P*_1_) tended to be narrower (*P* = 0.056), *P*_2_ tended to be higher (*P* = 0.068), *P*_3_ tended to be lower (*P* = 0.080), and *P*_4_ was significantly higher (*P* = 0.003) in the STZ group than in the CNT group. Since *P*_1_ and *P*_2_ showed opposite directional differences, *G*_max_ was not significantly different between the CNT and STZ groups. Empagliflozin significantly decreased *P*_3_ and *P*_4_, but post-hoc analysis only detected a significant difference in *P*_4_ between the CNT and STZ groups. The peripheral arc parameters did not differ significantly between the CNT and STZ groups or between BL and T3 (Fig. 4B). The intercept largely varied in the STZ group compared with the CNT group, partly because the higher minimum SNA in the STZ group yielded a larger estimation error of the intercept when extrapolating the regression line to 0% SNA. The operating-point AP was not significantly different between the CNT and STZ groups, whereas the operating-point SNA was significantly higher in the STZ group than in the CNT group (Fig. 4C). Empagliflozin did not significantly affect the operating-point AP or SNA.

**Fig. 5:**
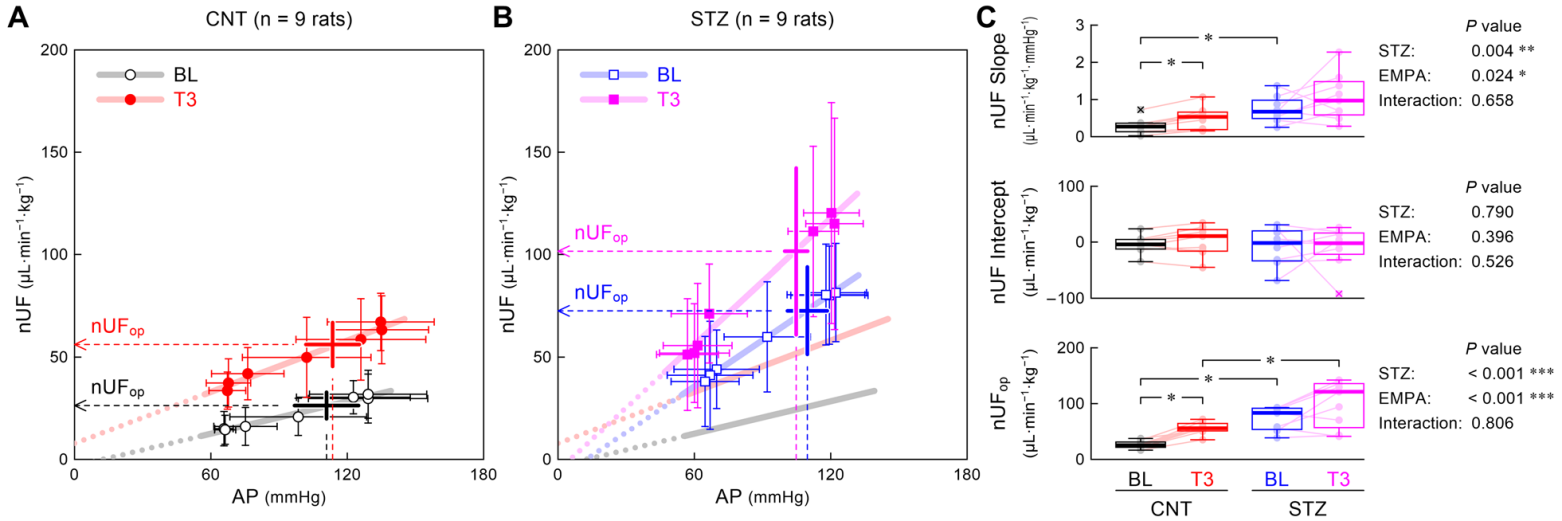
The relationship between arterial pressure (AP) and normalized urine flow (nUF) obtained in the control (CNT) (**A**) and streptozotocin (STZ) groups (**B**). The data points represent mean ± SD values during baseline (BL) and third period (T3) after empagliflozin administration. The pale thick lines represent the linear regression lines drawn over the mean data points. In panel **B**, the pale black and red lines are duplicated from panel **A**. The vertical dotted line shows the operating-point AP under each condition. The horizontal arrowhead shows the nUF corresponding to the operating-point AP (nUF_op_). Box-and-whisker plots showing the nUF slope, nUF intercept, and nUF_op_ (**C**). In the right, *P*-values are shown for the effect of STZ, the effect of empagliflozin (EMPA), and the interaction effect derived from a two-way analysis of variance with aligned rank transform. Post-hoc analysis was performed using the Wilcoxon signed-rank test (BL vs. T3) or Mann–Whitney test (CNT vs. STZ), with an appropriate correction of the significance level based on Holm’s method. *, **, and *** indicate *P* < 0.05, *P* < 0.01, and *P* < 0.001, respectively

Figure 5 shows the AP–nUF relationship during the stepwise changes in CSP. The nUF increased with AP in both the CNT (Fig. 5A) and STZ (Fig. 5B) groups. The STZ group had a significantly higher nUF slope than the CNT group, and empagliflozin significantly increased the nUF slope (Fig. 5C). The nUF intercept did not differ between the CNT and STZ groups, and empagliflozin did not significantly affect the nUF intercept. The STZ group had a significantly higher operating-point nUF than the CNT group, and empagliflozin significantly increased the operating-point nUF.

**Fig. 6:**
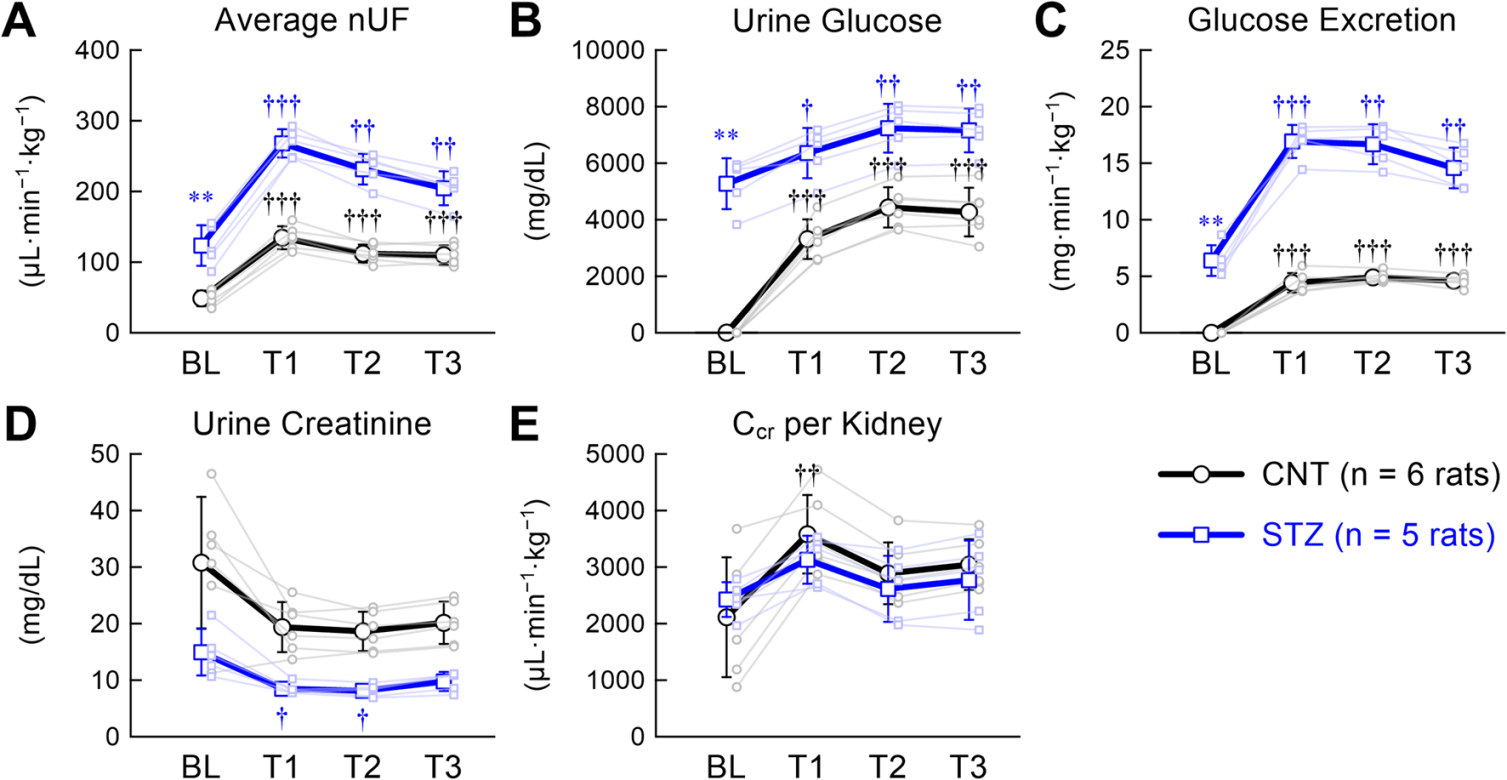
Changes in the averaged normalized urine flow (nUF) (**A**), urine glucose concentration (**B**), urine glucose excretion (**C**), urine creatinine concentration (**D**), and creatinine clearance (C_cr_) per kidney (**E**) obtained from six rats in the control (CNT) group and five rats in the streptozotocin (STZ) group. The data points show mean ± SD values during baseline (BL) and 1st (T1), 2nd (T2), and 3rd (T3) periods after empagliflozin administration. The data of the respective rats are shown in pale color. The BL values were compared between CNT and STZ rats using the Mann–Whitney test, with a significant difference indicated by ** (*P* < 0.01). Time-dependent changes of each parameter within each group were examined using a one-way analysis of variance with the Greenhouse–Geisser correction. Post-hoc analysis was performed against the BL value using Dunnett’s test, with significant differences indicated by † (*P* < 0.05), †† (*P* < 0.01), and ††† (*P* < 0.001)

The blood sample was obtained once at the end of the experiment in six CNT rats and five STZ rats; thus, the following data could be influenced by empagliflozin administration. The plasma noradrenaline concentration showed numerically higher values in the STZ group than in the CNT group, but the difference did not reach statistical significance [CNT: 77 (68–96) vs. 154 (112–181) pg/mL, *P* = 0.126]. The STZ group had a significantly higher plasma glucose concentration than the CNT group [CNT: 180 (152–194) vs. STZ: 351 (305–463) mg/dL, *P* = 0.004)], whereas the plasma creatinine concentration did not differ between the two groups [CNT: 0.39 (0.33–0.40) vs. STZ: 0.36 (0.31–0.42) mg/dL, *P* > 0.999)]. Compared with the CNT group, the average nUF during BL was more than twice as large in the STZ group (Fig. 6A). Empagliflozin significantly increased the average nUF within each group. In the CNT group, urine glucose concentration (Fig. 6B) and excretion (Fig. 6C) during BL were near zero. Empagliflozin significantly increased urine glucose concentration and excretion within each group. The urine creatinine concentration during BL tended to be lower in the STZ group than in the CNT group (*P* = 0.052, Fig. 6D). Although empagliflozin decreased the urine creatinine concentration in the CNT group as an overall effect (*P* = 0.033), the difference from BL was not detected by post-hoc analysis. Empagliflozin significantly decreased urine creatinine concentration during T1 and T2 compared with BL in the STZ group. The C_cr_ per kidney during BL did not differ significantly between the two groups (Fig. 6E). The mean lines of C_cr_ showed a similar trend for the two groups, but the time-dependent changes were significant only in the CNT group (*P* = 0.005) with a significant increase during T1 relative to BL in the post-hoc analysis.

## Discussion

### Effects of empagliflozin on Baroreflex function in STZ-induced DM rats

The STZ group had a significantly higher minimum SNA in the neural arc than the CNT group (Fig. 4A), indicating impaired sympathetic suppression in DM rats, which is consistent with previous studies [19, 20]. In the present study, only the percent suppression of SNA could be compared between the CNT and STZ groups because of the normalization of SNA. However, previous studies have shown that plasma noradrenaline levels were 2- to threefold higher in STZ-induced DM rats compared to control rats [21, 22], suggesting that the absolute SNA might have been higher in the STZ group than in the CNT group. The plasma noradrenaline levels measured at the end of the experiment were numerically higher in the STZ group than in the CNT group, supporting the presence of sympathetic activation in the STZ group even after empagliflozin administration. However, the magnitude of the absolute SNA suppression by empagliflozin remains unanswered due to the lack of baseline data on plasma noradrenaline concentration.

Empagliflozin significantly lowered the midpoint pressure and minimum SNA in the neural arc as a main effect (Fig. 4A), which may explain a decrease in the midpoint pressure of the total arc (Fig. 3A). Several factors can be involved in the sympathetic suppression by empagliflozin. First, renal stress may be associated with sympathetic activation via an excitatory renal reflex [23, 24]. Empagliflozin relieves renal stress by reducing renal energy consumption [5], which could have attenuated the excitatory renal reflex. Second, empagliflozin hyperpolarizes the neurons in the rostral ventrolateral medulla and reduces their frequency of action potential in a brainstem-spinal cord preparation [6]. Third, empagliflozin relaxes resistance arteries in an in vitro preparation [25]. It is possible that the vessels at the carotid sinus baroreceptors became more easily distended, enhancing the baroreflex-mediated sympathetic suppression. However, the vasodilatory effect of empagliflozin was not large enough to cause a displacement of the peripheral arc toward lower AP, as seen in the cases of calcium channel blockers [26] and a soluble guanylate cyclase stimulator [27]. In the present experimental settings, the sympathetic system governs the HR due to vagotomy. A decreased minimum HR was apparent only in the STZ group (Fig. 3B), possibly because the minimum SNA was already too low for a significant minimum HR reduction in the CNT group.

Although not well quantified in the fitted parameters of the neural arc, the SNA in some STZ rats showed nadirs in the CSP midrange (Fig. 1B). Empagliflozin decreased the SNA and AP in the CSP midrange in the STZ group (Fig. 2B). This non-uniform effect of empagliflozin possibly relates to the two fiber types in the baroreflex afferent pathway: A and C fibers. The A-fiber baroreflex governs the sympathetic regulation in a normal pressure range, whereas the C-fiber baroreflex contributes to sustained sympathetic suppression in the high input pressure range [28, 29]. Although both A- and C-fiber axons show morphometric alterations in STZ-induced DM rats [30], the lack of sustained sympathetic suppression in the high CSP range indicates a loss of function of the C-fiber baroreflex in the STZ group. In contrast, the A-fiber baroreflex might have retained a functional capacity to reduce the SNA and AP in the CSP midrange, which was enhanced after empagliflozin administration.

Despite statistically significant changes in several baroreflex parameters, the overall sympathoinhibitory effect of empagliflozin was modest, rendering no significant change in the operating-point SNA or AP (Fig. 4C). As a reference, central antihypertensives, such as rilmenidine [31] and clonidine [32], showed sympathetic suppression that significantly decreased operating-point SNA and AP under similar experimental conditions.

### Effects of empagliflozin on urine output function in STZ-induced DM rats

The nUF increased linearly with the mean AP in both groups (Fig. 5). The STZ group had a significantly higher nUF slope than the CNT group during BL, which was in contrast with the lower nUF slope in GK diabetic rats in our previous study [7]. The STZ group had a much higher urine glucose concentration during BL (Fig. 6B) than GK rats [214 (20–1209) mg/dL, *n* = 7], leading to glucose osmotic diuresis [33]. A previous study reported that glomerular filtration rate (GFR) increased in moderately hyperglycemic rats and decreased in severely hyperglycemic rats [34]. In the present study, C_cr_ during BL did not differ significantly between the CNT and STZ groups, indicating that GFR might not have been much different between both groups.

Empagliflozin significantly increased urine glucose excretion with increased nUF (Fig. 6). During T3, the STZ group had an averaged nUF of approximately 200 μL·min^−1^·kg^−1^ (12 mL·h^−1^·kg^−1^), which was twice the sum of the infusion rate of Ringer’s lactate solution and anesthetic mixture. Despite the negative fluid balance, the peripheral arc did not change significantly before and after empagliflozin administration in the STZ group (Figs. 2B,  4B). The maintenance of the peripheral arc after empagliflozin administration was consistent with the acute effect of empagliflozin observed in GK diabetic rats [7]. Compared with loop diuretics, SGLT2 inhibitors can retain intravascular fluid volume because of a differential volume regulation between interstitial and intravascular fluid volumes [35, 36]. Increased renal vascular resistance after empagliflozin administration, via the activation of tubulo-glomerular feedback, may also maintain AP against the negative fluid balance [37].

### Clinical implication

Although the STZ model represents only acutely developed DM, we include a discussion on the use of SGLT2 inhibitors in heart failure because there is a close link between DM and heart failure in clinical settings. DM increases the risk of heart failure by 2- to threefold [38]. DM can worsen heart failure, while heart failure can impair glucose metabolism through mechanisms such as hypoperfusion and congestion of the pancreas and liver [39]. An epidemiological study reported in 2015 indicates that approximately 24% of patients with heart failure in general population and 40% of those in hospitalized population have DM [40]. Although the cardioprotective effect was initially observed in patients with DM [1], SGLT2 inhibitors exert a beneficial effect on chronic heart failure regardless of DM [41, 42]. Currently, using SGLT2 inhibitors early following an acute myocardial infarction is investigated [43, 44]. Understanding the acute hemodynamic effect of empagliflozin may become more important considering the early use of the drug. In our previous studies using GK diabetic rats [7] and rats with chronic myocardial infarction [37], empagliflozin preserves sympathetic AP regulation despite a significantly increased urine output. The present study on STZ-induced DM rats reinforces the previous finding. It should be kept in mind, however, that BS levels and neurological disorders observed in clinical settings would be quite different from those in the STZ-induced DM model.

### Limitations

Because we only examined the acute effects of empagliflozin under anesthetized conditions, the results may not be directly applicable to understanding any chronic effect of empagliflozin. As an example, 1-week treatment with empagliflozin reduced the exaggerated sympathetic response to lowering blood pressure in conscious diabetic rabbits [45]. Such a chronic effect of empagliflozin may not be detected by the present framework. Second, the vagal nerves were sectioned to obtain open-loop conditions for the carotid sinus baroreflex. Central vagal activation is impaired in STZ rats [46]. However, we could not evaluate whether empagliflozin affected the vagal control. Third, whether the observed changes in baroreflex function are a class effect of SGLT2 inhibitors remain unknown. Further studies using other SGLT2 inhibitors are required to answer this question.

## Conclusions

Empagliflozin showed significant acute effects on the baroreflex total arc and HR control in STZ-induced DM rats, which may have resulted from a sympathoinhibitory effect of empagliflozin on the neural arc. However, the sympathoinhibitory effect of empagliflozin was much weaker than that observed with central antihypertensives under similar experimental settings. The peripheral arc did not change significantly despite the significant increase in nUF after empagliflozin administration. Thus, empagliflozin preserved the overall sympathetic AP regulation in STZ-induced DM rats. The lack of a significant change in the peripheral arc may minimize reflex sympathetic activation, thereby enhancing a cardioprotective benefit of empagliflozin.

## Data Availability

The datasets obtained in the present experiment are available from the corresponding author upon reasonable request.

## Abbreviations

ANOVA: Analysis of variance
AP: Arterial pressure
BL: Baseline
BS: Blood sugar
C_cr_: Creatinine clearance
CNT: Control
CSP: Carotid sinus pressure
DM: Diabetes mellitus
DMSO: Dimethyl sulfoxide
GK: Goto–Kakizaki
HR: Heart rate
nUF: Normalized urine flow
SD: Standard deviation
SGLT2: Sodium–glucose cotransporter 2
SNA: Sympathetic nerve activity
STZ: Streptozotocin
UF: Urine flow
UV: Urine volume
